# Downregulation of *ORP3* Correlates with Reduced Survival of Colon Cancer Patients with Advanced Nodal Metastasis and of Female Patients with Grade 3 Colon Cancer

**DOI:** 10.3390/ijms21165894

**Published:** 2020-08-16

**Authors:** Pengfei Xu, Julia Richter, Annette Blatz, Fabian Gärtner, Roland Alberts, Anca Azoitei, Wycliffe Arika Makori, Sabine Meessen, Uwe Knippschild, Cagatay Günes

**Affiliations:** 1 Department of General and Visceral Surgery, Surgery Center, Ulm University Hospital, 89081 Ulm, Germany; pengfei.xu@uniklinik-ulm.de (P.X.); richter.julia86@googlemail.com (J.R.); Annette.Blatz@uniklinik-ulm.de (A.B.); Fabian.Gaertner@uniklinik-ulm.de (F.G.); Roland.Alberts@uniklinik-ulm.de (R.A.); 2 Department of Urology, Surgery Center, Ulm University Hospital, 89081 Ulm, Germany; anca.azoitei@uniklinik-ulm.de (A.A.); arikamakori@gmail.com (W.A.M.); Sabine.Meessen@uniklinik-ulm.de (S.M.)

**Keywords:** ORP3, OSBPL3, tumor suppressor, colon cancer, genome instability

## Abstract

Genome instability is an essential hallmark in tumor development, including colorectal cancer. We have recently identified the oxysterol binding protein-related protein 3 (ORP3), also known as oxysterol binding protein-like 3 (OSBPL3), as a novel ploidy-control gene, whose knock-out leads to aneuploidy induction and promotes tumor formation, indicating that ORP3 is a bona fide tumor suppressor protein. Here we analyzed expression of *ORP3* in a cohort (*n* = 206) of colon cancer patients in relation to patient survival. We show that low *ORP3* mRNA levels correlate with reduced survival of patients with advanced nodal metastasis (N2). While patient survival does not associate with grading when the whole cohort is evaluated, importantly, low *ORP3* mRNA levels associate with worse survival of female patients with grade 3 colon cancer. Similarly, low *ORP3* mRNA levels associate with worse survival of grade 3 colon cancer patients 70 years of age and younger while low *ORP3* mRNA levels seem to be beneficial for colon cancer patients with a T2 tumor size. Together, the data show that *ORP3* expression is downregulated during colon cancer progression, which correlates with reduced patient survival. Thus, *ORP3* mRNA levels may be a prognostic marker for better stratification of colon cancer patients.

## 1. Introduction

Colorectal cancer (CRC) characterized as a multifactorial and heterogeneous disease is the third most common cancer worldwide and the second deadliest in malignancy [1]. Several environmental components, as well as genetic risk factors, are known to be associated with CRC [2,3,4,5]. Whereas 75% are sporadic cases, 20% of the cases have a family history of CRC, and 3–5% of CRCs are hereditary, characterized by germline mutations linked to cancer-predisposition syndromes, among them the Lynch syndrome (1–3%) and familiar adenomatous polyposis (FAP) [6,7]. Especially, chromosomal instability, mismatch repair, and hypermethylation contribute to CRC development and progression. While chromosomal instability results in an unbalanced equilibrium between oncogenes and tumor suppressors, deficiency of deoxyribonucleic acid (DNA) mismatch repair (dMMR), especially of the *Mut L homolog1* (*MLH1)* or the *Mut S homolog 2* (*MSH2)*, contributes to an increase of errors within the genome and DNA hypermethylation and can lead to the reactivation or silencing of genes, among them proto-oncogene B-raf (*BRAF)* and *MLH1* [8,9,10,11]. Defective dMMR finally results in a strong mutator phenotype, indicated by microsatellite instability (MSI), which drives tumorigenesis. In MSI-driven tumorigenesis of CRC, positively, as well as negatively, selected driver gene mutations have been identified [12,13]. Approximately, 15% of CRC show high microsatellite instability (MSI-H) due to germline mutations in genes involved in DNA mismatch repair or somatic inactivation of the same signal pathway, often induced through hypermethylation of *MLH1*. Although it has been shown that, MSI-H CRCs represent a heterogeneous group, they exhibit several unique biologic characteristics when compared to microsatellite stable (MSS) colon cancer [14,15,16,17,18]. They all share some distinct histologic cancer features with high amounts of tumor-infiltrating lymphocytes [19]. In addition, Lynch syndrome patients exhibit a high risk of synchronous or metachronous tumors [20]. Prognostically, patients with hereditary non-polyposis colorectal cancer (HNPCC) have an increased overall survival compared to stage-matched sporadic CRCs [21,22].

The cancer stage at the time of diagnosis is most important for prognosis underlined by the fact that CRC patients with a localized stage exhibit a five-year survival rate of 90 % whereas the five-year survival rate of patients with metastasis amounts to 11%. Surgery, as well as chemotherapy, are still the most common treatment options for CRC. Although therapy improvements contributed to prolonged median survival for CRC patients, the prognosis for patients with advanced stages is still not satisfactory due to high apoptotic resistance and metastatic potential of their tumors. Therefore, there is a high interest to identify new targets for drug development for personalized therapy concepts with enhanced selectivity, efficacy, and reduced toxicity. In this context, Oxysterol binding protein-related protein 3 (ORP3), also referred to as oxysterol binding protein-like 3 (OSBPL3), could serve as a new target for drug development underlined by the finding that ORP3 expression correlates with benefits from a lomustine (CCNU)/bevacizumab combination treatment in a specific molecular subtype of gliomoblastoma [23]. This study provides evidence for a potential benefit of patient stratification using ORP3 as a molecular marker.

ORP3 is a cytosolic lipid-binding/transfer protein that is targeted to the plasma membrane by its pleckstrin–homology (PH) domain, while its FFAT motif (two phenylalanine in an acidic tract) targets it to the endoplasmic reticulum (ER) [24,25,26]. Previous studies revealed ORP’s function as scaffolds for the protein phosphatases, protein phosphatase 2 (PP2A), and haematopoetic protein tyrosine kinase (HePTP), regulating the activity of extracellular signal-regulated kinases (ERK), thereby indicating a role of ORPs in tumor cell signaling [27]. Members of this family have been shown to impact cell migration and adhesion. ORP3 was shown to interact with R-Ras [25,26], and to regulate cell adhesion [25]. Increasing evidence indicates putative roles of ORP family members in cancer [28,29]; this is summarized in [30]. However, direct analysis of its potential role in cancer has not been conducted. Recently, we have identified a role of ORP3 in ploidy-control [31]. Although the exact mechanisms how ORP3 contributes to ploidy-control is yet to be elucidated, we could show that knock-down or loss of ORP3 induced genome instability and promoted tumorigenesis in vitro and in vivo, respectively [31,32]. Importantly, *Orp3* knockout mice develop B-cell lymphoma indicating a tumor suppressor function of ORP3 [32].

Since a role of ORP3 in colon cancer has not been addressed in detail so far, its role for survival of colon patients was characterized in the present study. The evaluation of the whole colon cancer cohort (*n* = 206) indicates that low *ORP3* mRNA levels associate with worse survival of patients with advanced nodal metastasis (N2). While patient survival does not associate with grading when the whole cohort is evaluated, importantly, low *ORP3* mRNA levels associate with worse patient survival in female patients with grade 3 colon cancer. Similarly, low *ORP3* mRNA levels associate with worse survival of grade 3 colon cancer patients under 70 years, while low *ORP3* mRNA levels seem to be beneficial for colon cancer patients with T2 tumor size.

In summary, in colon cancer, high *ORP3* levels may serve as a survival marker in combination with N2 status. In addition, there is a significant association for better survival for females and patients under 70 years with higher *ORP3* mRNA levels among grade 3 colon cancer patients.

## 2. Results

### 2.1. ORP3 mRNA Levels Are Downregulated in Colon Cancer

**Figure 1:**
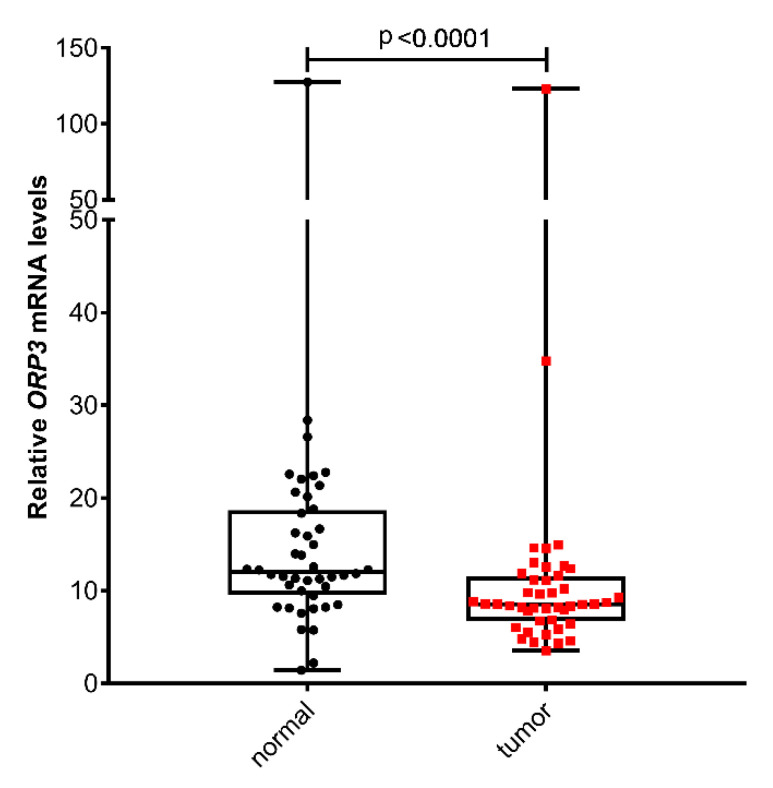
*ORP3* mRNA levels are downregulated in the tumor tissue of human colorectal cancer (CRC) patients. *ORP3* mRNA levels were determined in samples derived from matched normal and tumor tissues of the same patients in a cohort of 44 patients. Box plot analysis show the group comparison of relative *ORP3* RNA expression in normal and tumor tissues of CRC patients. (Wilcoxon test). Black dots represent normal tissue samples and red dots represent tumor tissue samples.

To test the possible relevance of the newly identified ploidy-control gene ORP3 for colon cancer we firstly aimed to determine its mRNA levels in a set of matched normal and tumor samples of 44 colon patients (31 males and 13 females) with a median age of 65 years (minimum: 29 years/maximum: 93 years). Whereas the group sizes of the different UICC stages are almost similar (UICC I:5, UICC II: 13, UICC III: 12, and UICC IV: 14), distribution by gender and tumor grade (grade 2:33, grade 3:9, grade 4:2) resulted in dissimilar groups. Box plot analysis of ORP3 mRNA levels of these 44 patients revealed that *ORP3* expression is statistically significant downregulated in tumor samples in comparison to matched normal tissue (*p* = 0.0001) (Figure 1).

### 2.2. Significance of ORP3 mRNA Levels for Patient’s Survival

To evaluate the significance of *ORP3* mRNA levels for patients’ survival we used a cohort of CRC patients (*n* = 206, Table 1, see Materials and Methods for details) and determined *ORP3* mRNA levels by RT-qPCR. Patients were recruited constitutively in the time period from 2003 until 2014.

### 2.3. Description of Study Population

The clinical and histopathological parameters of the colon cancer patient cohort are listed in Table 1. Altogether, 206 colon cancer patients (110 males, 96 females) with a median age of 70.41 years (range 29.81–89.67 years) and a median survival of 48.12 months (range 0.33–175.49 months) were considered in all analyses. Whereas gender ratio, group sizes of Union for International Cancer Control (UICC) stages, as well as ratios of tumors with and without invasion to lymph nodes are nearly similar within the respective subgroups, distribution by tumor grade leads to dissimilar groups with 135 patients in grade 2 group and 54 patients in grade 3 group, whereas grade 1 and 4 groups only encompasses 12, and 5 patients, respectively.

The ratio of metastasizing tumors to non-metastasizing tumors is 1:2.6. There are no statistically significant differences regarding median *ORP3* RNA levels within groups of gender, histological grade, tumor localization, and lymph node invasion (*p* = 0.666, *p* = 0.323, *p* = 0.878, *p* = 0.789, respectively; Table 1). Whereas the detected differences in *ORP3* RNA levels in the different UICC stages did not reach statistical significance (*p* = 0.055), the differences within the metastasis groups were statistically significant (*p* = 0.033). Furthermore, statistically significant differences in the median age of the patients were detected for the follow up (*p* = 0.001), the tumor localization (*p* = 0.014) and the lymph node groups (*p* = 0.032). Furthermore, the median survival for the follow-up (*p* = 0.001), the UICC stage (*p* = 0.001), the lymph nodes (*p* = 0.001) and metastasis (*p* = 0.001) groups were significantly different (Table 1).

**Figure 2:**
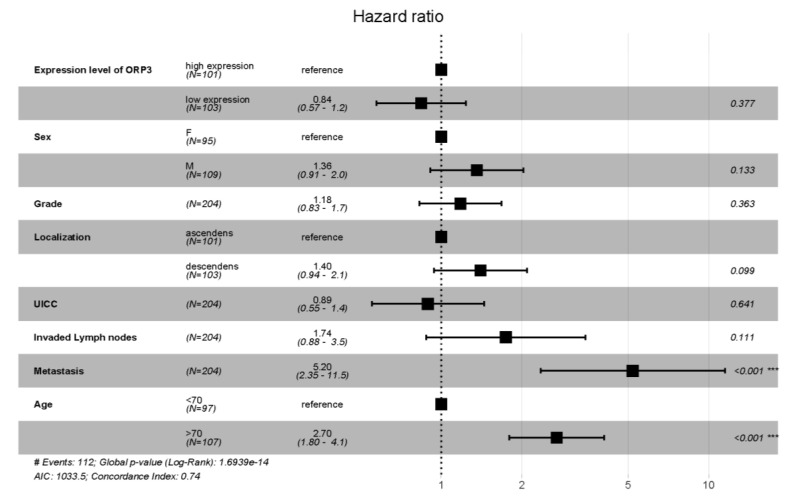
Hazard ratio calculated using a cox proportional-hazards analysis of the combined cohort. Hazard ratios > 1 indicate an increased risk of dying. While hazard ratios < 1 indicate a beneficial outcome for the patient. *p*-values of each individual factor based on the multivariate analysis is depicted on the right of the figure with the values: *** indicates *p* < 0.001.

This univariate analysis of the cancer patients’ cohort shows an influence of several parameters on patient’s survival. Due to the multivariate nature of this data set, a cox proportional-hazards analysis was conducted to cope with the potential of several covariables potentially affecting the prognosis of the patient (Figure 2). The multivariate analysis excludes the two patients with carcinoma in situ (CIS), ending up with *n* = 204. Additionally, the factor “expression level of *ORP3*” was introduced based on an expression threshold of the median *ORP3* RNA expression (RQ = 0.0082). This median was used to subdivide the samples into low (RQ < 0.0082) and high (RQ ≥ 0.0082) expression of *ORP3*. Furthermore, the patient’s cohort was tested for correlation between the different variables. The focus was put on correlation between the gene level of *ORP3* and N status, T status, grade and age. However none of the parameters age (correlation coefficient = 0.092, *p* = 0.187), N status (correlation coefficient = −0.015, *p* = 0.829), T status (correlation coefficient = −0.030, *p* = 0.669), and grade (correlation coefficient = 0.059, *p* = 0.397) correlates with the level of *ORP3* (Figure S3).

### 2.4. Survival Analysis in Relation to ORP3 mRNA Levels

Kaplan–Meier survival analyses were performed to evaluate the relevance of *ORP3* RNA levels for patient’s survival, comparing their survival rates with low and high *ORP3* RNA levels in the tumor samples.

**Figure 3:**
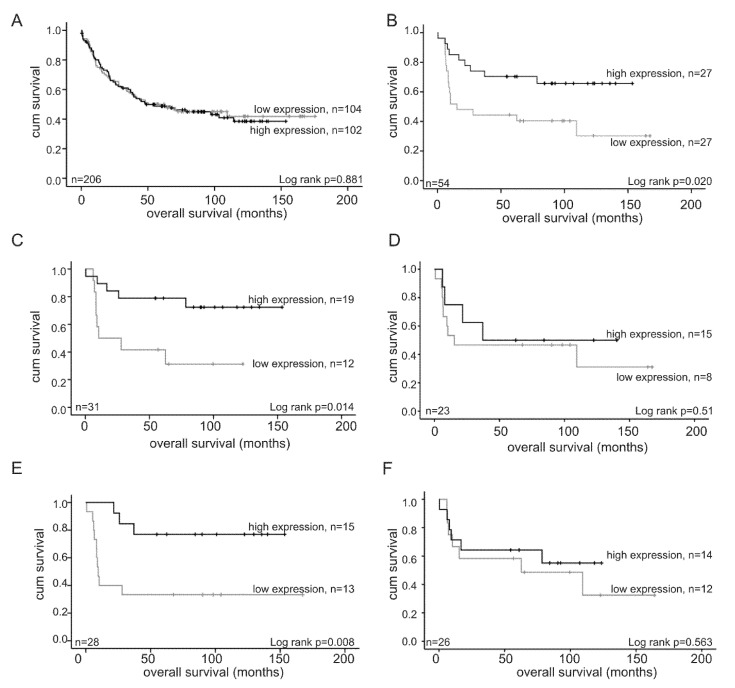
Impact of *ORP3* mRNA levels on prognosis of colon cancer patients. (**A**) *ORP3* mRNA levels in the whole cohort of colon cancer patients in correlation to patient´s survival, (**B**) *ORP3* mRNA levels in grade 3 human colorectal patients in correlation to patient’s survival; (**C**,**D**) *ORP3* mRNA levels in grade 3 tumors in correlation to female (**C**) and male (**D**) colon cancer patient’ survival; (**E**,**F**) *ORP3* mRNA levels in grade 3 human colorectal patients in correlation to patient’s age, (**E**) patients younger than 70 years old and (**F**) patients older than 70 years old. *ORP3* mRNA levels were determined by quantitative gene expression analysis in the whole cohort and in the subgroup of patients with grade 3 colon cancer. Patient’s cumulative survival was plotted against *ORP3* mRNA levels. The median value was used to group patients with high and low *ORP3* expression. Statistical analysis: Exploratory data analysis was performed using IBM SPSS Statistics 25 (SPSS Inc., Armonk, NY, USA). For statistical analysis of overall survival, Kaplan–Meier estimations were created and a significance was tested using log-rank test. *p*-values < 0.05 were considered statistically significant. No correction for multiple testing was done. Black: high *ORP3* expression levels, grey: low *ORP3* expression levels.

There was no significant difference in patient’s survival in relation to *ORP3* mRNA levels for the entire cohort (*p* = 0.881; Figure 3A). However, patients with grade 3 tumors expressing high *ORP3* mRNA levels showed significantly higher survival rates compared to patients with low *ORP3* mRNA levels (*p* = 0.020, Figure 3B). Importantly, patient´s survival was gender specific. Whereas high *ORP3* mRNA levels in grade 3 tumors significantly correlated with better survival rate of females, this was not the case for males with grade 3 tumors with high *ORP3* mRNA levels (females: *p* = 0.014, males: *p* = 0.516) (Figure 3C,D). Similarly, patient´s survival reached a higher significance when *ORP3* mRNA levels were analyzed in correlation to age-dependent survival rates of grade 3 patients. There is an age-specific survival benefit among the grade 3 group with high *ORP3* mRNA levels. Whereas patients below 70 years with grade 3 tumors and high *ORP3* RNA levels had significantly better overall survival (*p* = 0.008), no statistically significant correlation between *ORP3* mRNA levels and overall survival of over 70 years old patients was detected (*p* = 0.563) (Figure 3E,F). Of note, *ORP3* mRNA levels of grade 2 patients did not significantly correlate with patient survival (Supplementary Figure S1). No predication for patient´s survival in correlation to *ORP3* mRNA levels was possible for patients with grade 1 and grade 4 tumors due to low group numbers (*n* = 12 and *n* = 5, respectively). Similarly, there was no significant correlation between *ORP3* mRNA levels and patient´s survival in relation to the different UICC stages, UICC I (*p* = 0.218), UICC II (*p* = 0.198), UICC III (*p* = 0.798) and UICC IV (*p* = 0.988), respectively (Supplementary Figure S2).

**Figure 4:**
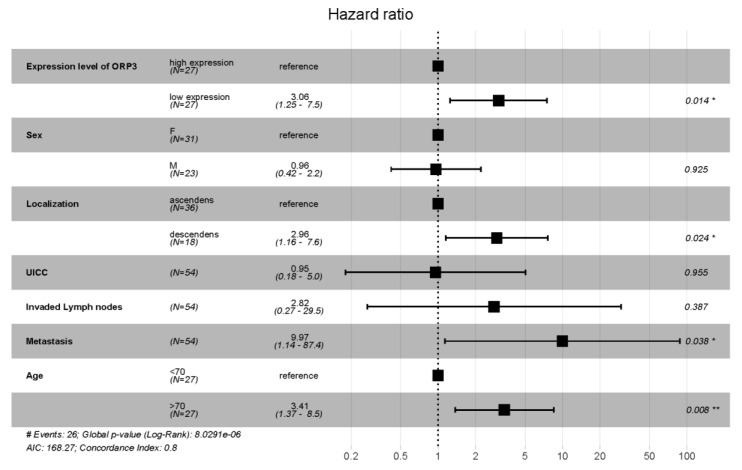
Hazard ratio calculated using a cox proportional-hazards analysis of patients with a grade 3 tumor. The factor grade was, therefore, masked from this analysis. Hazard ratios > 1 indicate an increased risk of dying. While hazard ratios < 1 indicate a beneficial outcome for the patient. *p*-values of each individual factor based on the multivariate analysis is depicted on the right of the figure with the values: * indicates *p* < 0.05, ** indicates *p* < 0.01.

A hazard ratio was also calculated for the whole cohort using a cox proportional-hazards analysis. The graphical evaluation as well as the calculated values show that the mRNA expression level of *ORP3* is not a predictive gene for the survival of the patient, if the whole patients’ cohort is analyzed. However, several other factors like high age and metastasis turn out to a risk for the patient´s survival. These results are in accordance with the univariate analysis depicted in Table 1. Importantly, however, the prognostic feature of *ORP3* mRNA levels for patient survival was confirmed by a multivariate analysis and cox proportional-hazards analysis when patients with grade 3 tumor were evaluated (Figure 4).

The multivariate analysis of the data set presented in Figure 4, containing only patients with a grade 3 tumor confirms the results acquired with a univariate analysis (Figure 3B). Patients with a grade 3 tumor and a low expression of *ORP3* mRNA (RQ < 0.0082) show a 3.05 times higher risk of death. The calculated values for each parameter are shown in the Supplementary Table S1.

**Figure 5:**
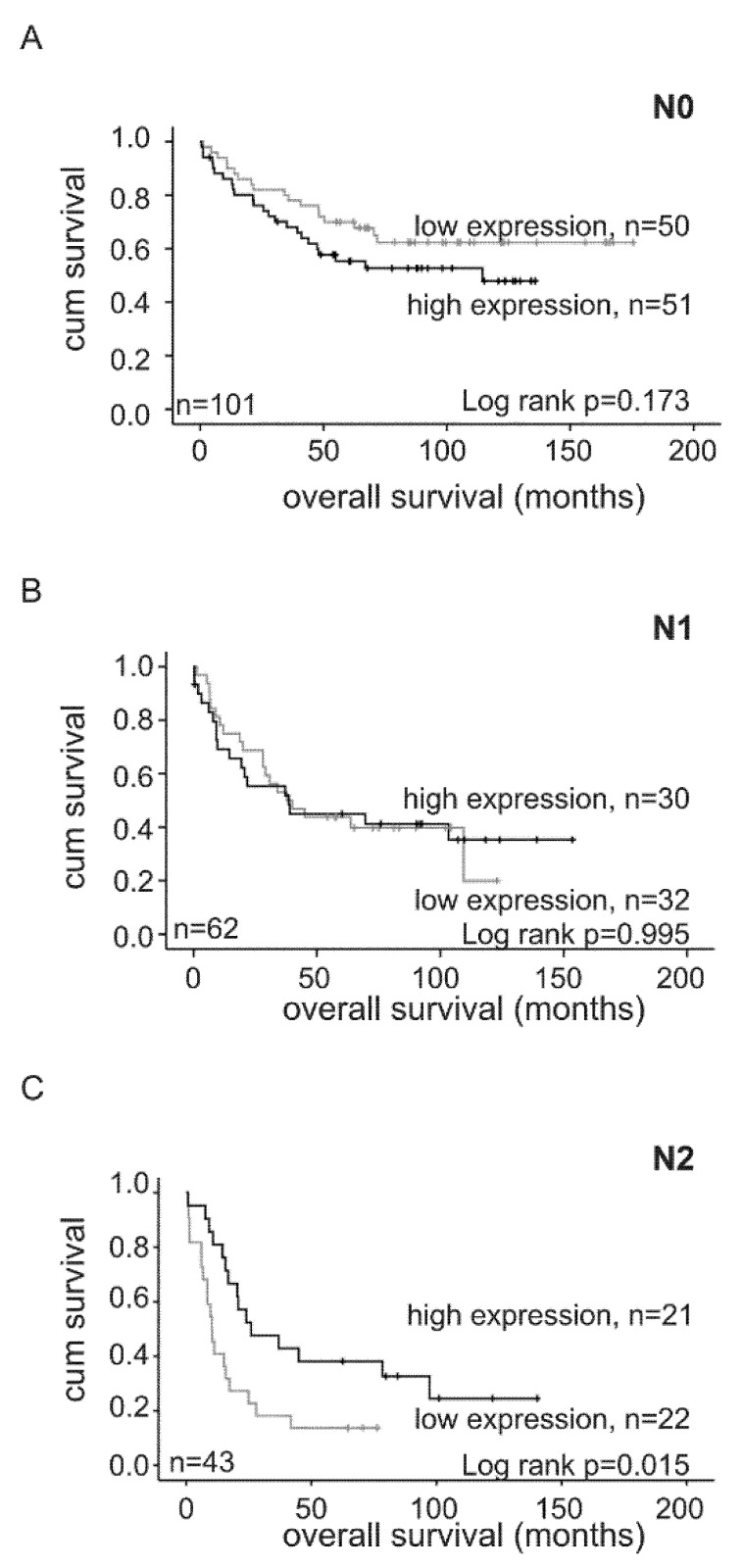
Low *ORP3* mRNA levels associate with reduced survival of advanced nodal metastasis of colon cancer patients. Relative quantification of *ORP3* RNA expression in N0 (low *ORP3* expression: 24 female, 26 male and high *ORP3* expression: 25 female, 26 male) (**A**), N1 (low *ORP3* expression: 14 female, 18 male and high *ORP3* expression: 15 female, 15 male) (**B**), and N2 (low *ORP3* expression: 9 female, 13 male and high *ORP3* expression: 9 female, 12 male) (**C**) classified tumors of colon cancer patients was performed by qRT-PCR using *ORP3* specific primers. HPRT and *β-ACTIN* were used as reference genes. Kaplan–Meier survival plots were generated using IBM SPSS Statistics 25. Black: high *ORP3* expression levels; grey: low *ORP3* expression levels.

Another interesting observation was made when *ORP3* levels were analyzed in correlation to lymph-node metastasis in the whole group. Importantly, patients classified as N2 nodal metastasis with high *ORP3* mRNA levels had significantly better survival rates in comparison to patients with low *ORP3* mRNA levels (*p* = 0.015), whereas *ORP3* mRNA levels did not impact survival of patients of the N0 (*p* = 0.173) and N1 subgroups (*p* = 0.995) (Figure 5). Of note, there are no sex-specific differences among the groups (see legend to Figure 5).

**Figure 6:**
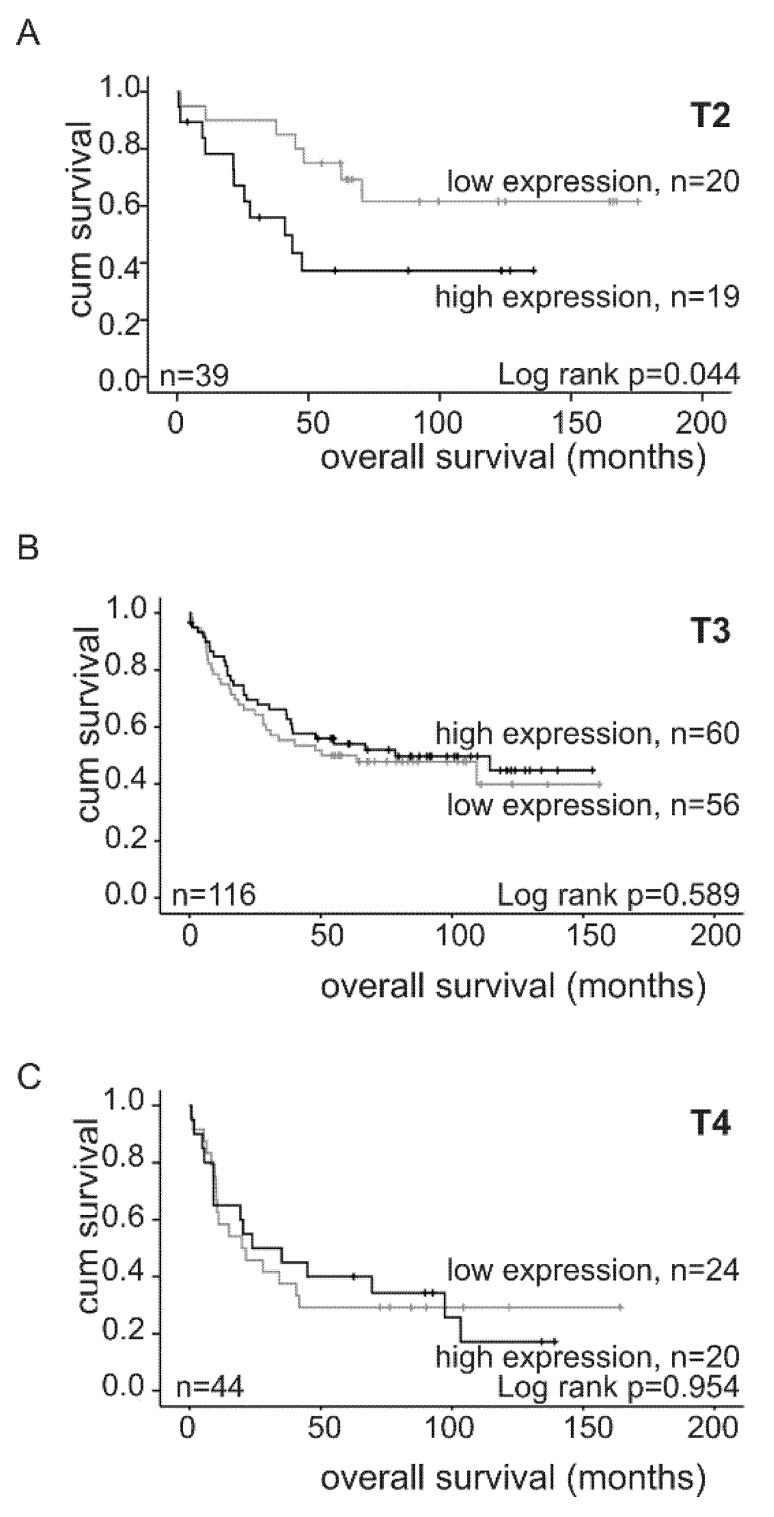
Influence of *ORP3* RNA expression on survival of colon patients with T2, T3, or T4 tumors. Relative quantification of *ORP3* RNA expression in T2 (low *ORP3* expression: 9 female, 11 male and high *ORP3* expression: 8 female, 11 male) (**A**), T3 (low *ORP3* expression: 22 female, 34 male and high *ORP3* expression: 33 female, 27 male) (**B**), and T4 (low *ORP3* expression: 12 female, 13 male and high *ORP3* expression: 7 female, 13 male) (**C**) tumors of colon cancer patients was performed by qRT-PCR using *ORP3* specific primers. HPRT and β-ACTIN were used as reference genes. Kaplan–Meier survival plots were generated using IBM SPSS Statistics 25.

On the other hand, reduced *ORP3* mRNA levels seemed to be beneficial for the survival of patients with small tumor size: while patients, whose tumor size was classified as T2, had significantly increased survival rates when *ORP3* RNA levels were low (*p* = 0.044) (Figure 6). No significant correlation between *ORP3* mRNA levels and survival rates of patients with T3 and T4 tumors could be observed. These differences were not sex-specific (see legend to Figure 3).

## 3. Discussion

The incidence and progression of colon cancer in humans has been shown to result from accumulation of genetic changes, accompanied by chromosomal instability (CIN). CIN, mostly characterized by increased aneuploidy, is a hallmark of cancer cells that goes along with the acquisition of defects in chromosomal segregation, by deregulated expression of various oncogenes and tumor suppressor factors and mutations in DNA damage response genes. However, the full composition of genes underlying aneuploidy remains incompletely described and the exact molecular mechanisms of carcinogenesis in colon carcinomas are still not fully understood. In addition, patient stratifications using molecular and histological markers are required for more efficient therapy of patients with colon cancer. In the current study, we addressed the potential impact of ORP3 on colon cancer to evaluate the prognostic benefit based on complete long-term follow-up data regarding tumor progression and overall survival. *ORP3* was identified in a genetic screen as a novel ploidy-control gene and its down-regulation induced aneuploidy and promotes tumor formation [31,32].

The role of ORP3 in colon cancer has not been addressed in detail so far. Of note, a recent study which combines proteomic and genomic analysis on colon cancer [33] revealed deregulation of ORP3 in 6.6% of colon cancer patients (4.72% mutations and 1.89% amplifications), as determined by the cBio Cancer Genomics Portal [34,35], supporting the idea that ORP3 may contribute to colon cancer initiation and/or progression. The molecular mechanisms how ORP3 may contribute to colon cancer remain to be elucidated. Interestingly, it was shown that the nuclear receptor, the liver receptor homologue 1, a member of nuclear receptor of subfamily 5 group A (LRH-1 or NR5A2), which acts upstream of ORP3 in liver hepatocytes and promotes non-alcoholic fatty liver disease by activating de novo lipogenesis via ORP3 [36], is a novel prognostic marker in colon cancer patients [37,38,39]. Although a direct regulation of ORP3 by LRH-1/NR5A2 in colon cancer was not demonstrated so far, it is conceivable to assume that ORP3 expression may be modulated by LRH-1/NR5A2 during colon cancer initiation and/or progression.

The measured values of *ORP3* mRNA expression in the tumor tissue describe a snapshot at the time of diagnosis after primary oncological resection. The study shows that patients with advanced lymph node metastasis (N2) and low *ORP3* mRNA levels exhibit reduced survival probability compared to patients of the same group but high *ORP3* mRNA levels. Moreover, in female patients and patients under 70 years of age with grade 3 tumors, high *ORP3* mRNA levels correlate with an increased overall survival. On the other hand, high *ORP3* levels correlate with worse survival in combination with smaller tumor size (T2) in colon cancer. In summary, our data show clear evidence that the altered expression of *ORP3* may be involved in the pathogenesis and progression of colon cancer. These data are in line with the potential tumor suppressive function of ORP3. Of note, ORP3 was shown to interact with R-Ras, a Ras-related cell signaling factor, which controls Ras signaling that is known to be one of the most often deregulated pathways in colon cancer [13,40,41,42]. In fact, we demonstrated that loss of *ORP3* expression activates Ras signaling and promotes tumor formation in *Orp3* knockout mice [32]. The *Orp3* knockout mice primarily developed B-cell leukemia, probably due to high *Orp3* mRNA expression in hematopoietic stem cells of C57BL/6 mice (C. Günes, unpublished results). Whether *Orp3* knockout promotes colon cancer initiation and progression needs to be elucidated in future studies. In addition, potential association of RAS mutations with ORP3 mutations and/or expression levels need to be elucidated in future studies.

Taken together, *ORP3* mRNA levels may help to improve stratification of patients with grade 3 colon cancer for an improved therapy option. In this line, it was shown that *ORP3* expression correlates with benefit from CCNU/bevacizumab combination treatment in a specific molecular subtype of glioma [23]. This study provides evidence for a potential benefit of patient stratification using ORP3 as a molecular marker, although we have to admit that the sample size is low in specific subgroups (i.e., grade 3 tumor patients with gender and age discrimination). Questions remain towards the relationship of patient survival with altered *ORP3* levels and specific patient subgroups. We have previously shown that the knock-down and the knockout of *ORP3* induces aneuploidy, promoting tumorigenesis. It seems conceivable to speculate that increased genome instability due to lower ORP3 levels accelerates tumor malignancy and is disadvantageous for survival of patients with advanced tumor progression (grade 3) or nodal metastasis (N2). Further prospective analyses of the relationships between the expression of *ORP3* and the course of the disease in colon carcinoma are useful in order to demonstrate the importance of ORP3 as a potential marker for predicting the prognosis in colon carcinoma.

## 4. Materials and Methods

### 4.1. Human Tumor Tissue

The cohort encompasses 206 patients suffering from colon cancer who were operated in the Department of General and Visceral Surgery of the University Hospital Ulm between 2003 and 2014. Patients were constitutively recruited for the study but only in the case that they had given their consent for collecting patient´s blood, tissue and clinical data for this study prior to surgery. The patients were informed verbally and in writing about the planned measures using a separate information sheet. Patients were also informed that they could withdraw their consent at any time and that their personal data would be treated with strict confidentiality and in compliance with data protection regulations. Exclusion criteria were no informed consent, withdraw of patient´s consent, and age under 18 years. The study was performed with the permission of the independent local ethics committee of the University of Ulm (approvals 211/2002, 16 December 2002, and 268/2008, 15 December 2008).

Tumor tissues from 206 patients stored in our tissue bank were used. Routine pathological analyses from all tissue samples collected during operation were performed and the following variables were considered for further analysis: sex, median survival, local tumor stage (according the Union for International Cancer Control, UICC [43,44], histological grade, localization, lymph node invasion, and metastasis.

### 4.2. RNA Extraction, cDNA Synthesis and Determination of cDNA Purity

Total RNA was isolated from frozen tumor tissue sections of colon cancer patients using the RNeasy Mini Kit (Qiagen, Hilden, Germany) according to the manufacturer’s protocol. RNA was extracted in 30 µL of RNase free H_2_O and stored at −80 °C. In total, 1 µg of total RNA was transcribed into cDNA using the AffinityScript cDNA Synthesis Kit (Agilent Technologies, Santa Clara, CA, USA) according to the manufacturer’s instructions. cDNA samples were kept at −20 °C until they were used for PCR. All PCR reactions were performed in thermal cycler (LabCycler SensoQuest, Göttingen, Germany) in a total volume of 25 µL (quantitative) or 50 µL (preparative) containing 1× polymerase-specific buffer, 0.25 mM dNTP mix, 100 ng of template DNA, 100 nM forward and reverse primers and 2 U DNA polymerase. (Thermo Fisher Scientific, Schwerte, Germany). For verifing cDNA quality and genomic DNA contamination the β-ACTIN primer pairs β-ACTIN_for (GGC ATC CTC ACC CTG AAG TA)/β-ACTIN-rev (GTC AGG CAG CTC GTA GCT CT) and β-ACTIN_I (CGA GCA GGA GAT GGC CAC TGC)/β-ACTIN_E (GTG AGC TCT CTG GGT GCT GGG), as well as Taq polymerase (5 PRIME, Hilden, Germany) were used. Cycling conditions were as following: initial denaturation 3 min at 94 °C, denaturation (40 s at 94 °C), annealing (40 s at 62 °C), elongation (1 min at 72 °C) 35 cyles, and final elongation 5 min at 72 °C. Experiments were done in duplicate. Results are shown as ∆Ct values.

### 4.3. Determination of ORP3 mRNA Levels by Polymerase Chain Reaction (PCR) in Tumor Samples of 206 Colon Cancer Patients

All PCR reactions were performed in a total volume of 25 µL (quantitative) or 50 µL (preparative) containing 1x polymerase-specific buffer, 0.25 mM dNTP mix, 100 ng of template DNA, 100 nM forward and reverse primers and 2 U DNA polymerase. (Thermo Fisher Scientific, Schwerte, Germany). For checking cDNA quality and genomic DNA contamination the β-ACTIN primer pairs β-ACTIN_for (GGC ATC CTC ACC CTG AAG TA)/β-ACTIN-rev (GTC AGG CAG CTC GTA GCT CT) and β-ACTIN_I (CGA GCA GGA GAT GGC CAC TGC)/β-ACTIN_E (GTG AGC TCT CTG GGT GCT GGG), as well as Taq polymerase (5 PRIME, Hilden, Germany) were used. Quantitative gene expression of *OSBPL3 (ORP3)* was performed using the LC480 cycler (Roche Applied Science, Mannheim, Germany), QuantiFast SYBR Green PCR Kit (Qiagen, Hilden, Germany), and QuantiTect Primer Assay (Hs_OSBPL3_1_SG Cat.no. QT00084070; Qiagen, Hilden, Germany) according to the manufacturer’s protocol. Primers for β-*ACTIN* (Hs_ACTB_2_SG Cat.no. QT01680476, Qiagen; Hilden, Germany) and *HPRT* (Hs_HPRT_1_SG Cat.no. QT00059066; Qiagen, Hilden, Germany) were used as endogenous controls. The efficiency of all three primer pairs was tested and resulted in the following efficiencies: *HPRT* = 103%, β-*ACTIN* = 101%, and *OSBPL3 (ORP3)* = 109%. Furthermore, in order to exclude primer dimers and to guarantee reaction specificity, melting points were analyzed after amplification. The experiments were carried out using the following protocol: initial denaturation phase (15 min, 95 °C), PCR cycle (45 times: 15 s denaturation at 95 °C, 30 s annealing at 55 °C, 30 s elongation at 72 °C), melt phase (15 s at 95 °C, 1 min at 65 °C, continuous acquisition during temperature ramp up to 97 °C). Experiments were done in duplicate. Results are shown as ∆Ct values.

### 4.4. Determination of ORP3 mRNA Levels by RT-qPCR in Matched Normal and Tumor Tissue of 44 Colon Cancer Patients

Following primer pairs were used to quantify *ORP3* and *HPRT1*, respectively: ORP3-F: 5′-GTCATCCGCCCTAGCACAAAA and ORP3-R: 5′-AGAGACTCGGCATGGATTCTG; HPRT1-F: 5′- TGAGGATTTGGAAAGGGTGT and HPRT1-R: 5′- GAGCACACAGAGGGCTACAA. Quantitative PCR (qPCR) was performed using the iQ SYBRgreen super mix (Bio-Rad; 170–8880) by Applied Biosystem 7300 real-time PCR system. Experiments were performed as three technical replicates. Results are shown as ∆Ct values. Cycling conditions were as above.

### 4.5. Statistical Analyses

Exploratory data analysis was performed using IBM SPSS Statistics 25 (SPSS Inc., Armonk, NY, USA). Exploratory data were depicted as median (minimum/maximum). For statistical analysis of overall survival, a Kaplan–Meier estimation was created, and significance was tested using log-rank test. Group comparisons were performed by applying Wilcoxon test. p values < 0.05 were considered statistically significant. No correction for multiple testing was done. Correlation analysis was performed using the Spearman–Rho correlation. For each analysis, the correlation coefficient was displayed in addition to the *p*-value (two-sided). The graphs were edited by using CORELDRAW Version 12 (Corel Corporation, Ottawa, Canada).

Multivariate analysis was performed using R4.0.0 (R Core Team, 2020) using the following packages: survival (3.2–3) [45], survminer (0.4.8) [46] and dplyr (1.0.0) [47]. The full reproducible code is depicted as part of the Supplementary Table S2.

## 5. Conclusions

In this study, we show that low *ORP3* mRNA levels correlate with reduced survival of colon cancer patients with advanced nodal metastasis (N2). In addition, the study revealed that low *ORP3* mRNA levels associate with worse survival of female patients with grade 3 colon cancer. Moreover, we found an age-dependent correlation for patient survival, as low *ORP3* mRNA levels associate with worse survival of grade 3 colon cancer patients 70 years of age and younger. In summary, the data show that *ORP3* expression is downregulated during colon cancer progression, which correlates with reduced patient survival. We conclude that *ORP3* mRNA levels may be a prognostic marker for better stratification of colon cancer patients.

## Figures and Tables

**Table 1:** Clinical parameters of colon cancer patients’ cohort. Clinical parameters of the colon cancer patients in regard to UICC stage, tumor grade, sex, and localization of tumor within the colon referring to median age, survival and expression level of *ORP3*. Expression levels are calculated as relative quantification (RQ) using hypoxanthine phosphoribosyltransferase (HPRT) and β-ACTIN as housekeeping genes by using LightCycler^®^480 Multiplate Analysis Software. Abbreviations: cis: carcinoma in situ; min: minimum; max: maximum; *ORP3*: oxysterol binding protein-related protein 3, also known as oxysterol binding protein-like 3 (*OSBPL3*), UICC: Union for International Cancer Control, n.d.: not determined.

	Total	Median *ORP3* mRNA Levels [RQ] (Min/Max)	Median Age [Years] (Min/Max)	Median Survival [Months] (Min/Max)
Follow-up		*p = 0.526*	p = 0.001 *	p < 0.001 *
alive	92	0.008 (0.001/0.019)	67.32 (29.81/88.23)	91.05 (0.46/175.49)
dead	114	0.008 (0.0005/0.1088)	73.55 (39.21/89.68)	17.02 (0.33/114.41)
Sex		*p = 0.666*	*p = 0.293*	*p = 0.102*
M	110	0.008 (0.0005/0.1088)	70.37 (29.81/88.58)	38.91 (0.39/175.49)
F	96	0.008 (0.0006/0.1029	70.87 (39.69/89.67)	56.01 (0.33/164.77)
Stage (UICC)		*p = 0.113*	*p = 0.073*	p < 0.001 *
I	37	0.00811 (0.0016/0.0191)	75.37 (45.31/89.67)	64.57 (0.52/175.49)
II	52	0.008273 (0.0020/0.0248)	70.82 (32.34/87.95)	67.75 (1.18/164.05)
III	58	0.009395 (0.0009/0.1088)	69.00 (40.17/88.23)	64,16 (0.33/153.48)
IV	57	0.007249 (0.0005/0.1029)	67.58 (29.81/87.59)	14.50 (0.65/140.23)
cis	2			
Stage (UICC) female		p = 0.005 *	*p = 0.504*	p < 0.001 *
I	19	0.0077 (0.0015/0.0148)	74.28 (51.42/89.68)	64.57 (1.15/164.77)
II	24	0.0096 (0.0024/0.0165)	68.82 (40.35/87.95)	74.54 (1.18/156.09)
III	29	0.0107 (0.0027/0.0299)	71.55 (42.13/88.23)	69.57 (0.33/153.49)
IV	23	0.0069 (0.0006/0.1029)	68.32 (39.70/86.49)	12.01 (1.31/81.09)
cis	1			
Stage (UICC) male		*p = 0.936*	*p = 0.123*	*p = 0.002*
I	18	0.0084 (0.0038/0.0191)	77.65 (45.31/88.58)	57.48 (0.52/175.49)
II	28	0.0073 (0.0019/0.0248)	73.05 (32.34/84.79)	67.75 (4.51/164.04)
III	29	0.0081 (0.0009/0.1087)	67.34 (40.17/84.67)	62.50 (0.39/134.17)
IV	34	0.0080 (0.0005/0.033)	66.37 (29.81/87.59)	15.42 (0.66/140.23)
CIS	1			
Grade		*p = 0.323*	*p = 0.360*	*p = 0.092*
1	12	0.008211 (0.0006/0.0165)	74.99 (39.69/88.09)	54.39 (3.91/164.77)
2	135	0.008082 (0.0005/0.0325)	70.17 (40.17/89.67)	47.53 (0.39/175.49)
3	54	0.008264 (0.0019/0.1088)	69.19 (29.81/88.23)	61.74 (0.33/167.37)
4	5	0.015794 (0.0042/0.0248)	75.56 (58.98/87.95)	4.93 (0.76/30.36)
Localization		*p = 0.245*	p = 0.041 *	*p = 0.557*
descendens	104	0.0079 (0.0005/0.0313)	68.20 (39.21/88.58)	54.58 (0.39/175.49)
ascendens	102	0.0085 (0.0006/0.1087)	72.86 (29.81/89.68)	40.90 (0,32/167.37)
Lymph nodes		*p = 0.789*	p = 0.032 *	p = 0.001 *
not invaded	101	0.008204 (0.0016/0.0248)	73.74 (32.33/89.67)	64.57 (0.53/175.49)
invaded	105	0.008124 (0.0005/0.1088)	67.87 (29.81/88.23)	27.96 (0.33/153.49)
Metastasis		p = 0.033 *	*p = 0.128*	p < 0.001 *
negative	149	0.008587 (0.0010/0.1088)	71.54 (32.33/89.67)	64.64 (0.33/175.49)
positive	57	0.007250 (0.0005/0.1029)	67.58 (29.81/87.58)	14.50 (0.66/140.23)
Total	206	0.008126 (0.0005/0.1088)	70.41 (29.81/89.67)	48.12 (0.33/175.49)

* *p* < 0.05 in the Mann–Whitney U Test or Kruskal–Wallis Test.

## Supplementary Materials

Supplementary materials can be found at https://www.mdpi.com/1422-0067/21/16/5894/s1. Supplementary Figure S1: ORP3 mRNA levels of grade 2 patients did not significantly correlate with patient´s survival. No predication for patient´s survival in correlation to ORP3 mRNA levels was possible for patients with grade 1 and grade 4 tumors due to low group numbers (*n* = 12 and *n* = 5, respectively); Supplementary Figure S2: Impact of ORP3 RNA expression on prognosis of the different UICC subgroups of colon cancer patients. Kaplan–Meier plot display the overall survival of patients with UICCI, UICC II, UICC III, and UICC IV tumors of colon cancer patients, divided according to the relative ORP3 RNA expression quantified by qPCR using specific primers. HPRT gene served as reference gene. Graphs were created using IBM SPSS Statistics 25; * *p* ≤ 0.05; Supplementary Figure S3: The figure shows the box plots representing average expression of ORP3 in separate groups of N0–N2, T1–T4 and grades 1–4; Supplementary Table S1: Output of the cox proportional-hazards analysis for the whole cohort. Results for the code coxph(formula = Surv(Time, Outcome) ~ factor(“Expression level of ORP3”) + Sex + Grade + factor(Localization) + UICC + “Invaded Lymph nodes” + Metastasis + Age, data = Cohort). Significance levels are shown as follows: * indicates *p* < 0.05, ** indicates *p* < 0.01, *** indicates *p* < 0.001; Supplementary Table S2: Output of the cox proportional-hazards analysis of patients with a grade 3 tumor. Results for the code: coxph (formula = Surv(Time, Outcome) ~ factor(“Expression level of ORP3”) + Sex + factor(Localization) + UICC + “Invaded Lymph nodes” + Metastasis + Age, data = Grade3). Significance levels are shown as follows: * indicates *p* < 0.05, ** indicates *p* < 0.01, *** indicates *p* < 0.001.

## Abbreviations

ORP3	oxysterol binding protein-related protein 3
CRC	Colorectal cancer
FAP	familiar adenomatous polyposis
dMMR	DNA mismatch repair
MLH1	Mut L homolog1
MSH2	Mut S homolog 2
BRAF	Proto-oncogene B-raf
OSBPL3	oxysterol binding protein-like 3
ER	endoplasmic reticulum
UICC	Union for International Cancer Control
cis	Carcinoma in situ
CIN	Chromosomal instability
PP2A	Protein phosphatase 2
HePTP	hematopoietic protein tyrosine kinase
ERK	extracellular signal-regulated kinases
CCNU	lomustine
MDPI	Multidisciplinary Digital Publishing Institute
DOAJ	Directory of open access journals
TLA	Three letter acronym
LD	linear dichroism
DNA	deoxyribonucleic acid
HPRT	Hypoxanthine Phosphoribosyltransferase
MSI	microsatellite instability
MSI-H	high microsatellite instability
HNPCC	hereditary non-polyposis colorectal cancer
MSS	micro-satellite stable
PH	pleckstrin–homology
min	minimum
may	maximum
NR5A2	nuclear receptor of subfamily 5 group A
LRH-1	liver receptor homologue 1
