# Supplementary material for: Downregulation of ORP3 Correlates with Reduced Survival of Colon Cancer Patients with Advanced Nodal Metastasis and of Female Patients with Grade 3 Colon Cancer
Source: Int J Mol Sci. 2020 Aug 16;21(16):5894. doi: 10.3390/ijms21165894 (PMC7460621; doi:10.3390/ijms21165894)
Supplement: Supplementary file 1 [file ijms-21-05894-s001.pdf]

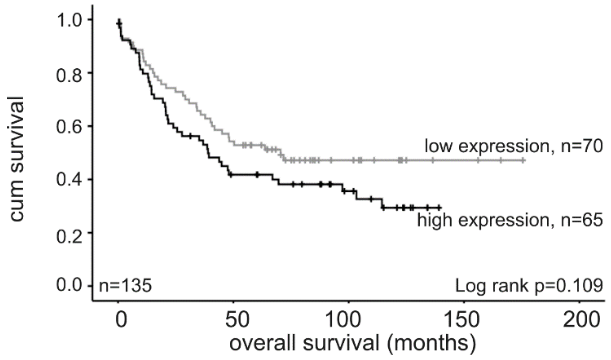

Supplemental Figure S1: *ORP3* mRNA levels of grade 2 patients did not significantly correlate with patient´s survival. No predication for patient´s survival in correlation to ORP3 mRNA levels was possible for patients with grade 1 and grade 4 tumors due to low group numbers (n= 12 and n=5, respectively).

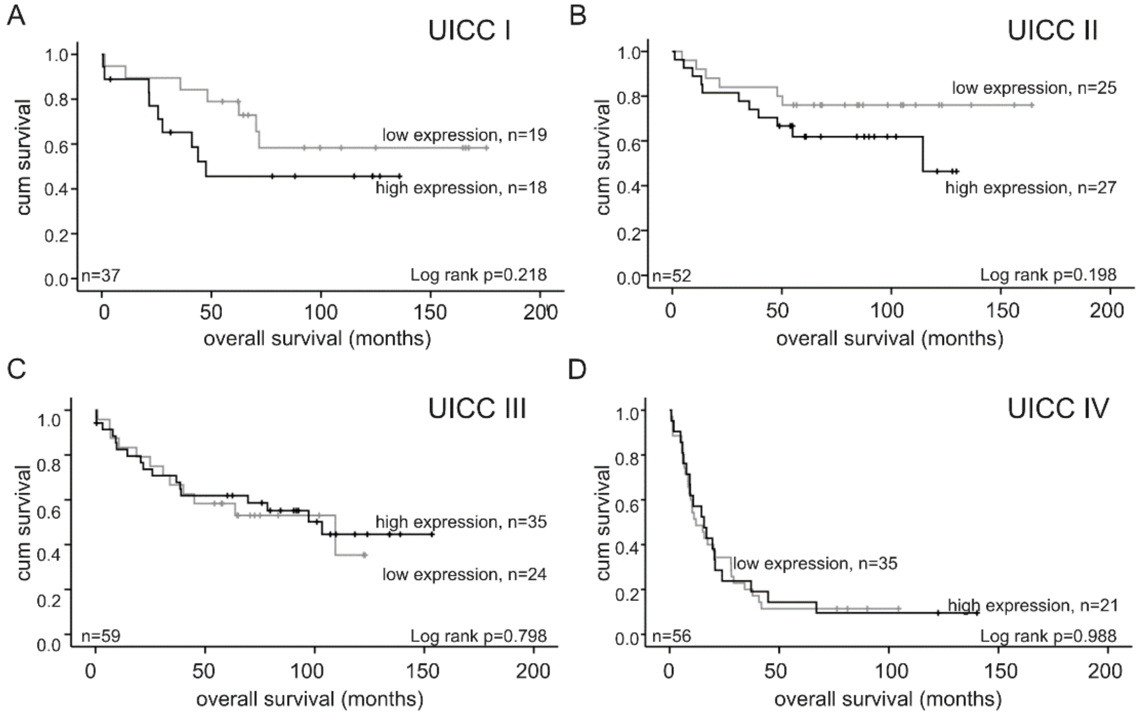

Supplemental Figure S2: Impact of ORP3 RNA expression on prognosis of the different UICC subgroups of colon cancer patients. Kaplan-Meier plot display the overall survival of patients with UICCI, UICC II, UICC III, and UICC IV tumors of colon cancer patients, divided according to the relative *ORP3* RNA expression quantified by qPCR using specific primers. *HPRT* gene served as reference gene. Graphs were created using IBM SPSS Statistics 25; *p ≤ 0.05.

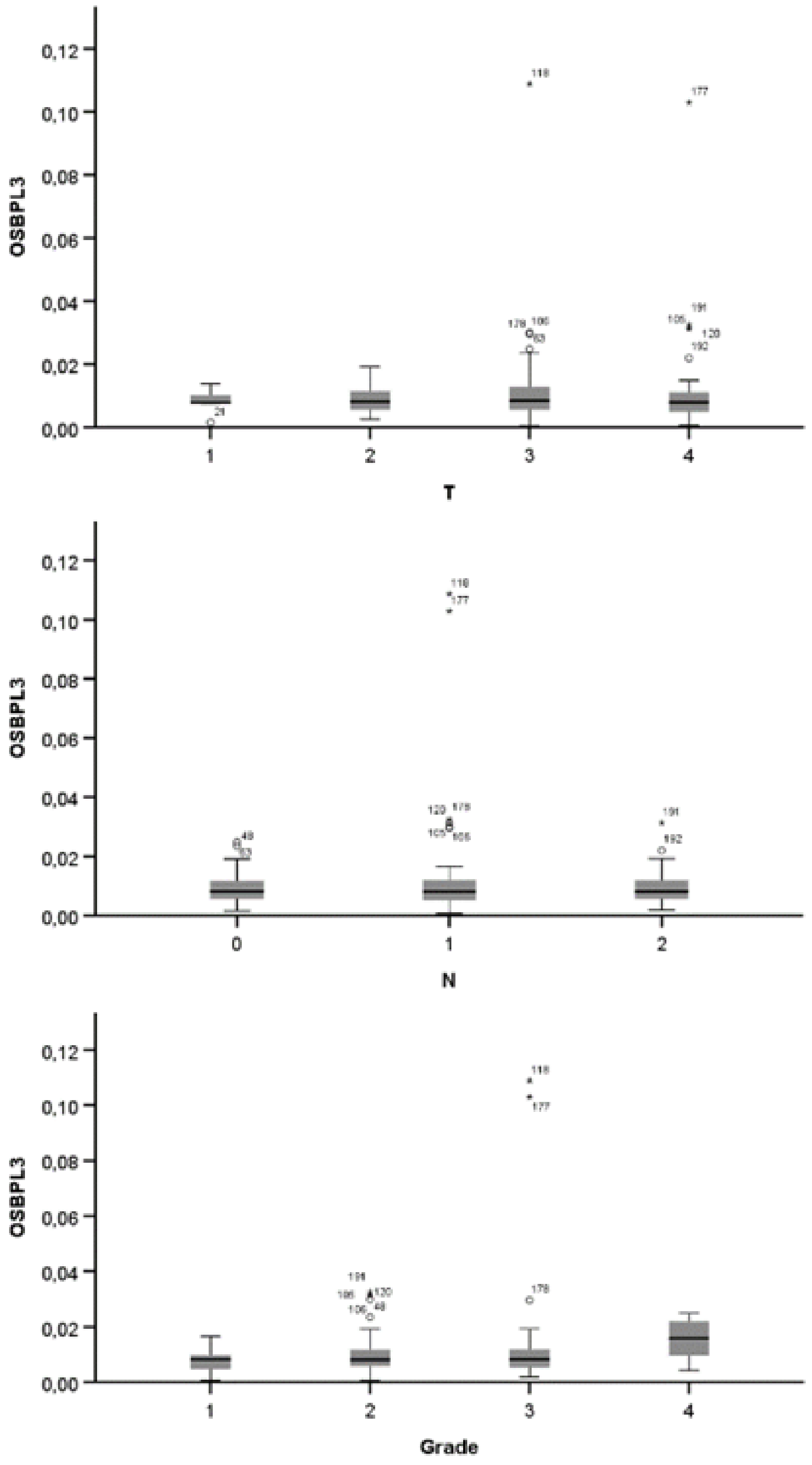

Supplemental Figure S3: The figure shows the box plots representing average expression of *ORP3* in separate groups of N0-N2, T1-T4 and grades 1-4.

Supplementary Table S1: Output of the cox proportional-hazards analysis for the whole cohort. Results for the code coxph(formula = Surv(Time, Outcome) ~ factor(`Expression level of ORP3`) + Sex + Grade + factor(Localization) + UICC + `Invaded Lymph nodes` + Metastasis + Age, data = Cohort). Significance levels are shown as follows: * indicates $p < 0.05$, ** indicates $p < 0.01$, *** indicates $p < 0.001$.

	coef	exp(coef)	se(coef)	z	Pr(>|z|)	Significance
Factor `Expression level of ORP3' low expression	-0,1728	0,8413	0,1955	-0,884	0,3768	
Sex Male	0,3068	1,3591	0,2044	1,501	0,1333	
Grade	0,1646	1,1789	0,1809	0,91	0,3629	
Factor (Localization) descendens	0,3385	1,4029	0,2051	1,651	0,0988	.
UICC	-0,115	0,8914	0,2464	-0,467	0,6408	
Invaded Lymph nodes	0,5565	1,7446	0,3496	1,592	0,1114	
Metastasis	1,6488	5,2008	0,4049	4,072	4,66e-05	***
Age>70	0,9938	2,7016	0,2077	4,784	1,72e-06	***

	exp(coef)	exp(-coef)	lower .95	upper .95
Factor `Expression level of ORP3' low expression	0,8413	1,1886	0,5735	1,234
Sex Male	1,3591	0,7358	0,9105	2,029
Grade	1,1789	0,8482	0,827	1,681
Factor (Localization) descendens	1,4029	0,7128	0,9386	2,097
UICC	0,8914	1,1219	0,5499	1,445
Invaded Lymph nodes	1,7446	0,5732	0,8792	3,462
Metastasis	5,2008	0,1923	2,3518	11,501
Age>70	2,7016	0,3702	1,798	4,059

Supplementary Table S2: Output of the cox proportional-hazards analysis of patients with a grade 3 tumor. Results for the code: coxph(formula = Surv(Time, Outcome) ~ factor(`Expression level of ORP3`) + Sex + factor(Localization) + UICC + `Invaded Lymph nodes` + Metastasis + Age, data = Grade3). Significance levels are shown as follows: * indicates $p < 0.05$, ** indicates $p < 0.01$, *** indicates $p < 0.001$.

	coef	exp(coef)	se(coef)	z	Pr(>|z|)	Significance
Factor `Expression level of ORP3' low expression	1,1192	3,06242	0,4567	2,451	0,01426	*
Sex Male	-0,03987	0,96091	0,42348	-0,094	0,92499	
Grade	1,08561	2,96125	0,48022	2,261	0,02378	*
Factor (Localization) descendens	-0,04767	0,95345	0,84907	-0,056	0,95523	
UICC	1,03609	2,81818	1,1974	0,865	0,38688	
Invaded Lymph nodes	2,29958	9,97	1,10742	2,077	0,03785	*
Metastasis	1,22645	3,4091	0,46529	2,636	0,00839	**
Age>70	1,1192	3,06242	0,4567	2,451	0,01426	*

	exp(coef)	exp(-coef)	lower .95	upper .95
Factor `Expression level of ORP3' low expression	3,0624	0,3265	1,2512	7,496
Sex Male	0,9609	1,0407	0,419	2,204
Grade	2,9612	0,3377	1,1553	7,59
Factor (Localization) descendens	0,9534	1,0488	0,1805	5,035
UICC	2,8182	0,3548	0,2696	29,458
Invaded Lymph nodes	9,97	0,1003	1,1378	87,364
Metastasis	3,4091	0,2933	1,3696	8,486
Age>70	3,0624	0,3265	1,2512	7,496
